# The Prevalence and Determinants of Hand and Face Dermatitis during COVID-19 Pandemic: A Population-Based Survey

**DOI:** 10.1155/2020/6627472

**Published:** 2020-12-05

**Authors:** Mohammed Saud Alsaidan, Aisha H. Abuyassin, Zahra H. Alsaeed, Saqer H. Alshmmari, Tariq F. Bindaaj, Alwa'ad A. Alhababi

**Affiliations:** ^1^Internal Medicine Department, College of Medicine, Prince Sattam Bin Abdulaziz University, Al-Kharj, Saudi Arabia; ^2^Dar Al Uloom University, Riyadh, Saudi Arabia; ^3^Prince Sattam Bin Abdulaziz University, Al-Kharj, Saudi Arabia

## Abstract

**Background:**

During the current COVID-19 pandemic, prevention is the key to limiting the spread of this disease. The frequent handwashing and use of sanitizers resulted in notable skin changes among some individuals. The aim of the study was to determine the prevalence and determinants of the new onset of dermatitis during the COVID-19 pandemic in a university population from Saudi Arabia.

**Methods:**

A cross-sectional study was conducted using a self-administered online questionnaire by sending an invitation link to students and employees of Prince Sattam Bin Abdulaziz University in June 2020. A chi-squared test was used to note differences regarding hand and face dermatitis.

**Results:**

Of the total 2356 participants, 34.8% reported skin changes or symptoms over hands, and 15.3% reported skin changes on their face during this pandemic. 88.7% of the participants reported a change in handwashing habits during the COVID-19 pandemic, and 62.2% of participants were not using any hand sanitizers before COVID-19 but began using them during the pandemic. There were significantly higher percentage of skin conditions in females (on hands (ScH): 42.6% and face (ScF):19.2%), individuals working in environments requiring frequent handwashing (ScH: 40.3% vs. ScF: 17.2%), those working in facilities where they have to interact with people during the pandemic (ScH: 41.1% vs. ScF: 18.7%), those encountering COVID-19 patients (ScH: 48.6% vs. ScF: 24.8%), those exposed to chemicals (ScH: 48.6% vs. ScF: 24.8%), and healthcare workers (ScH: 51.3% vs. ScF: 24.3%).

**Conclusion:**

It was found that during the pandemic, skin changes were common among the general population as well as among healthcare workers. The frequency of handwashing and the use of alcohol-based sanitizers were contributing factors for dermatitis. Although hygiene is an extremely important preventive measure in this pandemic, maintaining skin integrity is also vital. Appropriate knowledge and good practice can prevent dermatitis in this pandemic, with regular hydration of the skin being a key factor.

## 1. Introduction

An ongoing pandemic caused by the coronavirus disease (COVID-19) has been reported globally, with millions of cases and hundreds of thousands of deaths to date [1]. These numbers are increasing daily around the world. The spread of this deadly infection is from person to person through close contact—either directly through the inhalation of small droplets produced as a result of sneezing, coughing, or talking by infected persons or indirectly through contact with contaminated surfaces [2]. Currently, there is no effective antiviral treatment or vaccine available against this virus. The only way to prevent and reduce the rate of this infection is by using surface disinfectants, regularly washing hands, using hand sanitizers, covering the mouth and face with face masks, and using personal protective equipment (PPE) [3].

Evidence has indicated that countries that strictly followed guidelines of complete lockdown, physical distancing, and prevention strategies reported a reduced burden and fewer cases of COVID-19. Hand hygiene comes first in prevention strategies and for the reduction of hospital-acquired infection [4]. Hand hygiene with soaps and water or by using an alcohol-based sanitizer is the widely used method that is cheap, effective, and simple against COVID-19 [5]. The WHO emphasizes hand hygiene at the right times in a sufficient way in order to prevent the spread by 50% [3, 6].

Alcohol is commonly used in sanitizers as it possesses disinfectant and biocidal properties. These properties vary depending on the strength, type, and antiviral activity of the alcohol being used in sanitizers [7]. In 2017, the WHO developed two hand rub formulations for the prevention of enveloped viruses [8]. Antiviral and antibacterial activity of sanitizers increases with higher concentrations of alcohol mixed with other organic/inorganic acids [9, 10]. The efficacy of sanitizers in preventing the spread of many pathogens has been well documented [11]. However, precautions must be taken when frequently using sanitizers, as the excessive use of these agents may cause side effects. For instance, oils secreted by the sebaceous glands of the skin have inherent antiviral properties [12]. The continual use of alcohol-based hand sanitizers washes away these oils, leaving the skin dehydrated, which in turn results in fissures and erosion, allowing easy access to pathogens and increasing the risk of microbial infection [3]. The use of alcohol-based hand sanitizers markedly increased following the recommendations suggested by the Centers for Disease Control and Prevention (CDC) and WHO to fight against COVID-19 [13]. Consequently, this not only led to panic buying and overuse of sanitizers but also increased the supply of substandard products in the markets [14]. These products have limited antiviral activity, leading to the emergence and re-emergence of some microorganisms and dermatological problems [15].

The excessive use of soaps (bars or liquid) and detergents is also harmful. Frequent and prolonged handwashing causes a disruption of the lipid barrier (epidermal), resulting in increased skin sensitivity to any physical and chemical agents [16]. Although soaps and detergents are weak irritants, overzealous use of them causes cumulative irritant contact dermatitis and redness of the skin, mainly over the back of the hand and the webs of the fingers [16]. Other dermatological problems include contact cutaneous xerosis, flaking, allergic or irritant contact, dermatitis, and eczema [17, 18]. Some countries reported an increased incidence of skin problems during this pandemic [19–22]. In light of the aforementioned concerns and the increased incidence of hand eczema reported around the world, this study aims to identify the prevalence and associated factors of dermatitis during the COVID-19 pandemic in Saudi Arabia.

## 2. Materials and Methods

### 2.1. Study Design

A cross-sectional study was conducted in June 2020.

### 2.2. Study Setting and Study Population

A total of 2356 individuals participated in the study. An invitation link was sent to students and employees of Prince Sattam Bin Abdulaziz University, Al-Kharj, Saudi Arabia. Participants who accepted the invitation filled the online questionnaire. Individuals from both the general population and healthcare workers were included in the study. All the data were collected anonymously through SurveyHero online. The study design obtained the required ethical approvals of the ethics committee of the Prince Sattam Bin Abdulaziz University (PSAU/COM/RC/IRB/p/80). The consent to participate was a part of the questionnaire.

### 2.3. Data Collection Tools

The data were collected using the self-administered questionnaire.The questionnaire was administered in both Arabic and English versions and was translated back and forth by two bilingual translators. The questionnaire was developed after an in-depth literature review and was reviewed by two independent dermatologists. A pilot study of 30 participants was conducted, and the questionnaire was adjusted accordingly.

### 2.4. Statistical Analysis

A descriptive analysis was conducted. Mean/median and standard deviation (SD)/interquartile range (range) were reported for continuous variables, while frequency and percentages were calculated for categorical variables. The Shapiro–Wilk (S-W) tests were conducted for the variable age and showed a *p* value of 0.01, indicating that age variable was not normally distributed in our sample population. A chi-squared test was conducted to observe differences with respect to hand and face dermatitis. A *p* value of 0.05 or less was considered significant. Data are reported in tabular and graphical form. SPSS version 25 was used to conduct the analysis.

## 3. Results

Data from 2356 individuals were collected. Of the total number of participants, 55.9% were females, 76.4% were single, and the majority (93.0%) were Saudi nationals. The median age of participants was 21.00 (IQR: 18–26). 27.8% were employed or self-employed, and 56.1% were students. 64.5% of respondents had a monthly family income that was less than 10000 Saudi Riyals (Table 1).

When asked about their handwashing practices, 42.6% of the employees reported that they work in settings that require frequent handwashing, and 38.6% reported that they work in settings that necessitate encounters with customers. Additionally, 14.6% reported working in settings that require the use of irritating materials like chemicals, soaps, or other detergents. Only 4.5% of participants were working in settings in which there was direct contact with COVID-19 patients, and 6.9% were healthcare workers (Table 1). Moreover, 12% of participants had eczema, 9% had asthma, 2.3% had urticaria, 7.4% had rhinitis, 0.7% had rosacea, 19.7% had acne, and 5.8% reported having other skin conditions.

More than two-thirds (88.7%) of the participants reported that their handwashing habits changed during the COVID-19 pandemic. 42.2% reported washing their hands 3.5 times per day before the COVID-19 pandemic, but during the pandemic, the frequency of handwashing increased drastically 6–20 times for 70.4% of individuals. Nearly two-thirds (68.6%) reported washing their hands for less than 1 minute every time, while 27.4% reported washing it for 1–2 minutes. 82.6% reported using lukewarm water when washing their hands. Nearly half reported using antiseptic soaps for washing their hands, 53.4% reported using regular soaps, 52.0% reported using perfumed soaps, and 85% reported using liquid soaps (Table 2).

Regarding the usage of hand sanitizers, 87.6% reported that their habit of using hand sanitizers has changed during the pandemic. 62.2% were not using any hand sanitizers before COVID-19, but during the pandemic, 53.7% were using them 3–10 times per day, and 23.5% were using perfumed sanitizers. Of the total number of participants, 77% preferred handwashing to using hand sanitizers. 67.6% reported using hand sanitizers after coming into contact with any person or surface, while 21% were using it without getting in contact with anyone. In addition, 62% used it every time they entered their homes after being outside, and 48.5% reported using it before and after eating (Table 2).

Furthermore, 70.1% reported that they believed that excessive handwashing or the excessive use of sanitizers could cause skin problems. In regard to glove usage, 42.1% were not using any gloves, with 10.8% of participants being reported not using gloves because of skin problems. Out of those who did wear gloves, 35.6% used 1–2 pairs per day. With respect to face masks, 75.5% reported using surgical/medical face masks; 54.2% of the participants used the masks for half an hour to 2 hours; and 66.8% reported changing it 1–2 times per day.

**Figure 1:**
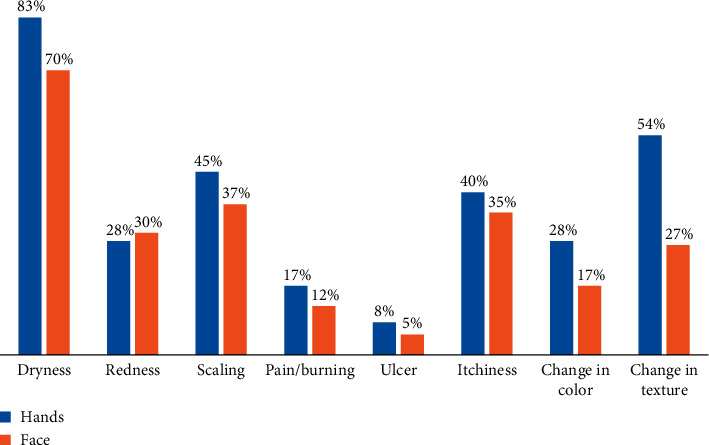
Skin conditions on hands and the face during the pandemic (*n* = 2356).

Of the total number of respondents, 34.8% (821 individuals) reported skin changes or symptoms during COVID-19, of whom 83.2% reported skin dryness, 54.2% reported changes in the texture, 45.4% reported scaling, 39.6% reported itchiness, 28.4% reported changes in skin color, 28.1% reported redness, and 17.4% reported pain/burning, while 7.6% reported skin ulcers (Figure 1). In order to alleviate these symptoms, 77.9% began to use moisturizers, 19.7% reported limiting handwashing and sanitizing, 7.2% consulted a doctor, and 5.6% used topical steroids. Only 29.8% reported improvements in skin changes.

**Figure 2:**
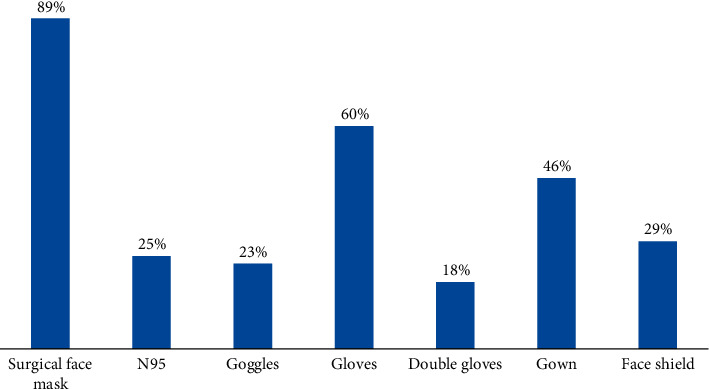
PPE used by healthcare workers during the pandemic (*n* = 163).

15.3% (360 individuals) reported skin changes on their faces during the pandemic. 4.9% of those with skin changes believed that they were due to the usage of face masks. However, the symptoms were mild for most (87.2%) of the patients. One hundred sixty-three of the participants were healthcare workers, and among them, 78.5% were working in hospitals (Figure 2).

There is a significantly higher prevalence of figures of skin conditions on patients' hands and face in the following demographics: females (skin condition on hands (ScH): 42.6% and skin condition on face (ScF): 19.2%), married individuals (ScH: 42.2% and ScF: 17.3%), those who were employed (ScH: 42.8% and ScF: 18.8%), individuals working in environments that require handwashing several times (ScH: 40.3% vs. ScF: 17.2%), those who were working in facilities where they have to interact with people during the pandemic (ScH:41.1% vs. ScF: 18.7%), those who were encountering COVID-19 patients (ScH: 48.6% vs. ScF: 24.8%), those working in facilities where they have to work with chemicals (ScH: 48.6% vs. ScF: 24.8%), and healthcare workers (ScH: 51.3% vs. ScF: 24.3%) [Table 1].

Skin conditions on hands were significantly more common among specific groups, including individuals who did not change their handwashing habits during the pandemic (37.1%), those who washed their hands several times per day (43.1%), those who washed more than 2 minutes (38.7%), or those who washed hands with cold water (38.9%). Skin conditions were also reported to be significantly more common among individuals who altered their usage of hand sanitizers during the pandemic (36.3%), those who used sanitizers several times per day (39.2%), those who used sanitizers with a greater alcohol concentration (46.1%), those who sanitized every one to two hours (40.8%), those who sanitized after coming from outside (36.4%), and those who sanitized before and after eating (38.7%) (Table 2).

Similarly, individuals who were using hand gloves (37.2%), who previously had eczema (55.8%), and who reported doing household work for more than 2 hours (37.7%) had significantly more skin conditions (58.3%). However, while skin conditions on the face were related to wearing facemasks, the duration of wearing facemasks and the number of facemasks changed per day did not affect skin conditions (Table 3).

## 4. Discussion

This population-based study was conducted to determine the prevalence of hand dermatitis in Saudi Arabia during the ongoing COVID-19 pandemic. In the study, most participants were Saudi nationals, young, females, single, and employed, while 7% were healthcare workers. Around two-fifths of the participants practiced frequent handwashing. The majority of participants reported that their handwashing habits changed, with their frequency of handwashing increasing from three times a day to 20 times a day during this pandemic. Two-third of the participants washed their hands for <1 min, while one-third washed them for 1–2 min every time. Half of the participants used soaps and water, while 77% used hand sanitizers. 35% of participants reported skin changes, with skin dryness being frequently presented. As a result of these changed washing and sanitizing habits among the Saudi population, 34.8% reported skin changes over hands and 15.1% over the face.

Dermatitis is an inflammatory response of the skin caused by allergens, irritant substances, or both [23]. As seen in this study, frequent handwashing altered the skin texture, with changes ranging from dryness to dermatitis. In this study, more than one-third reported skin changes, mostly skin dryness and altered skin texture, with some cases of redness, scaling, pain, itching, or even ulcers. A review by Cristina Beiu highlighted these potential adverse effects that can be caused by immoderate handwashing [17]. A study conducted in India found 16 new cases of hand eczema in the general population due to excessive hand hygiene practices [19]. Similarly, a Chinese study reported a 74.5% prevalence of hand eczema in healthcare providers [20], while another study reported a 90.2% incidence of eczema among German healthcare workers [21]. Moreover, a study conducted in Milan, Italy, reported an increased frequency of hand eczema [22].

It is also known that, in addition to sanitizers, the constant and prolonged use of soap water in humid environments causes the disruption of the skin's outer layer, which in turn increases the skin's permeability to various agents [24]. Furthermore, stress, atmospheric aspects, quarantine, and lockdowns increase the prevalence and severity of many disease including atopic dermatitis [25]. Wet work (working with one's hands in a wet environment for >2 hours a day, or washing hands >20 times a day) and gloves occlusion (using waterproof gloves for >2 hours) causes skin barrier impairment, which, when combined with exposure to soaps or sanitizers, can trigger irritant contact dermatitis [26]. This can also be seen in individuals who wear gloves for a prolonged time. Healthcare workers working in COVID-19 wards have to wear PPE for several hours and, hence, are susceptible to adverse skin reactions [27]. Other published studies have shown that the use of gloves can lead to the development of contact dermatitis [6, 28]. Likewise, another study showed that the odds of developing dermatitis increased three times with the use of >5 pairs of gloves [29]. It was also found that dermatitis was significantly prevalent in 37% of individuals who used gloves (>1 pair per day).

It was found that most of the skin changes (46%) were significantly present in participants who used sanitizers with an alcohol concentration of more than 60%. According to the WHO's recommendation, when one's hands are not visibly dirty or when soaps and water are not available, alcohol-based hand rubs (ABHRs) with an alcohol concentration of >60% are the most appropriate alternative [5]. Similarly, in another study, all patients who came for dermatologic consultation because of skin damage had been washing their hands >10 times a day and had been using alcohol-based gel [22]. Since coronavirus is enveloped in a lipid bilayer, an alcohol-based sanitizer is undoubtedly effective. However, its excessive use can create multiple problems if not controlled in a timely manner. The major problems are the existence of substandard products in the markets as well as the emergence of alcohol tolerance, antimicrobial resistance (AMR), opportunistic infection, and product toxicity [30–32].

Dermatitis is significantly more prevalent among the healthcare workers and individuals who have contact with COVID-19 patients in this study, as they follow strict hygiene practices and wear PPE for a longer period of time. Similarly, a study reported a 97% prevalence of general skin damage in healthcare workers caused by excessive hygienic measures [20]. Another study reported a 90.4% prevalence of acute hand dermatitis and a 14.9% prevalence of hand eczema among healthcare workers [21]. Recent published studies also reported high prevalence figures of eczema in healthcare workers [6, 33].

In this study, some of the participants also faced skin changes on their faces due to the excessive use of face masks and PPE. A recent study reported that 97% of the skin damage was due to enhanced protective measures, and these include 83% of the nasal bridge lesion [34]. Other studies reported similar findings in addition to reporting other dermatologic side effects like pressure injury, urticaria, dryness of the skin, allergic contact dermatitis, and aggravation of underlying dermatosis. In all of these conditions, occlusion and friction were the main contributing factors [35–38]. These results were more commonly found among healthcare workers, who wear protective gear for prolonged times [20].

There are some limitations to the study. Firstly, only an online survey was conducted, with no dermatologist confirming the diagnosis. Secondly, the study is cross-sectional, so although the majority of the participants reported skin changes after the excessive use of hand hygiene, a temporal relationship and causality cannot be concluded. This can be better evaluated through prospective studies. Furthermore, all the symptoms were self-reported; therefore, the chance of recall bias cannot be excluded. However, the major strength of the study is that it covers both the general population and healthcare workers, in addition to using a relatively large sample size. Although the study was conducted on a large sample size, it only included students and employees of Prince Sattam Bin Abdulaziz University; therefore, the results may not be applicable to the general population and further studies are needed.

## 5. Conclusion

In conclusion, this study found that during this pandemic, skin changes were common among the general population as well as healthcare workers. This is mainly due to excessive handwashing and the use of alcohol-based sanitizers. Changes in washing habits, the frequency (>10 times) and duration (>2 min) of handwashing, and sanitizer alcohol concentration (>60%) are also contributing factors. Although hygiene is a crucial preventive measure in this pandemic, maintaining skin integrity is also of the utmost importance. It is also known from previous studies that the disruption of the skin barrier can provide a route of entry for the coronavirus as the angiotensin-converting enzyme (ACE-2)—the cell receptor for COVID-19—is present in hair follicles, the epidermis, and the blood vessels of the skin [19]. Further, only 7.3% of those who developed skin changes looked for medical assessment during this pandemic. Proper awareness and sufficient practice can prevent skin changes during this pandemic, especially because dermatitis is easily preventable and manageable, with regular hydration of the skin. Also, educating the public about using over-the-counter medications–if needed–could also be a key factor to prevent further complications for those who cannot visit a physician during a pandemic.

## Figures and Tables

**Table 1:** Sociodemographics and hand hygiene practices during the COVID-19 pandemic, and the prevalence of skin conditions (*n* = 2354).

Variables	Total *n* = 2354 (%)	Skin conditions on hand (*n* = 821)		Skin conditions on face (*n* = 360)	
		Yes (%)	*p* value	Yes (%)	*p* value
*Gender*					
Male	1038 (44.1)	260 (25.0)	<0.001	107 (10.3)	<0.001
Female	1318 (55.9)	561 (42.6)	—	235 (19.2)	

*Marital Status*					
Single	1842 (78.2)	604 (32.8)	<0.001	272 (14.8)	0.19
Married	514 (21.8)	217 (42.2)	—	88 (17.1)	

*Employment*					
Employed	656 (27.8)	281 (42.8)	<0.001	123 (18.8)	0.004
Housewife/Student	1700 (72.2)	540 (31.8)	—	237 (13.9)	

*Healthcare worker*					
Yes	163 (6.9)	95 (58.3)	<0.001	54 (33.1)	<0.001
No	2193 (93.1)	726 (33.1)	—	306 (14.0)	

*Handwashing∗*					
Yes	1003 (42.6)	404 (40.3)	<0.001	173 (17.2)	0.022
No	1353 (57.4)	417 (30.8)	—	187 (13.8)	

*Encounter with people having COVID-19∗*					
Yes	910 (38.6)	374 (41.1)	<0.001	170 (18.7)	<0.001
No	1446 (61.4)	447 (30.9)	—	190 (13.1)	

*Encounter with COVID-19 patients∗*					
Yes	105 (4.5)	51 (48.6)	0.003	26 (24.8)	0.006
No	2251 (95.5)	770 (34.2)	—	334 (14.8)	

*Use of chemicals∗*					
Yes	345 (14.6)	177 (51.3)	<0.001	84 (24.3)	<0.001
No	2011 (85.4)	644 (32.0)	—	276 (13.7)	

**Table 2:** Factors leading to skin conditions on hands during the COVID-19 pandemic (*n* = 821).

Variables	Total *n* = 2354 (%)	Skin conditions on hand (*n* = 821)	
		Yes (%)	*p* value
*Handwashing habit changed*			
Yes	2090 (88.7)	46 (17.3)	<0.001
No	266 (11.3)	775 (37.1)	

*Handwashing*			
≤10 times	1338 (56.8)	382 (38.6)	<0.001
>10 times	1018 (43.2)	439 (43.1)	

*Washing time*			
<1 min	1617 (68.6)	535 (33.1)	0.030
1–2 min	646 (27.4)	250 (38.7)	
>2 min	93 (3.9)	36 (38.7)	

*Handwashing water*			
Luke warm	1945 (82.6)	693 (35.6)	0.003
Cold	231 (9.8)	58 (25.1)	
Hot	180 (7.6)	70 (38.9)	

*Type of soap*			
Regular	1257 (53.4)	428 (34.0)	0.385
Antiseptic	1099 (46.6)	393 (35.8)	

*Perfumed soap*			
Yes	1226 (52.0)	431 (35.2)	0.744
No	1130 (48.0)	390 (34.5)	

*Type of soap*			
Solid	353 (15.0)	116 (32.9)	0.396
Liquid	2003 (85.0)	705 (35.2)	

*Changed in hand sanitizer habit*			
Yes	2064 (87.6)	750 (36.3)	<0.001
No	292 (12.4)	71 (24.3)	

*Hand sanitizer per day*			
≤ 10 times	1864 (79.1)	628 (33.7)	0.022
>10 times	492 (20.9)	193 (39.2)	

*Alcohol concentration*			
<60%	191 (8.1)	77 (40.3)	<0.001
>60%	503 (21.3)	232 (46.1)	
Do not know	1662 (70.5)	512 (30.8)	

*Perfumed sanitizer*			
Yes	554 (23.5)	187 (33.8)	0.537
No	1802 (76.5)	634 (35.2)	

*Sanitizing when getting in contact*			
Yes	1593 (67.6)	602 (37.8)	<0.001
No	763 (32.4)	219 (28.7)	

*Every one or two hours*			
Yes	495 (21.0)	202 (40.8)	0.002
No	1861 (79.0)	619 (33.3)	

*After coming from outside*			
Yes	1473 (62.5)	536 (36.4)	0.043
No	883 (37.5)	285 (32.3)	

*Before and after eating*			
Yes	1143 (48.5)	442 (38.7)	<0.001
No	1213 (51.5)	379 (31.2)	

*Hand gloves/day*			
Do not wear	1093 (46.4)	351 (32.1)	0.010
>1 pair	1263 (53.6)	470 (37.2)	

*Stopped using gloves*			
Yes	254 (10.8)	149 (58.7)	<0.001
No/do not know	2102 (89.2)	672 (32.0)	
*Had hand eczema*			
Yes	355 (15.1)	198 (55.8)	<0.001
No	2001 (84.9)	623 (31.1)	

*Household work*			
≤2 hours/week	1502 (63.8)	499 (33.2)	0.028
>2 hours	854 (36.2)	322 (37.7)	

**Table 3:** Factors leading to skin conditions on the face during the COVID-19 pandemic (*n* = 821).

Variables	Total *n* = 2354 (%)	Skin conditions on face (*n* = 360)	
		Yes (%)	*p* value
*Face mask*			
Surgical	1779 (75.5)	291 (16.4)	0.019
Others	430 (18.3)	47 (10.9)	
Do not use	147 (6.2)	22 (15.0)	

*Face mask hours*			
</ = 2 hours	1277 (54.2)	187 (14.6)	0.645
>2 hours	713 (30.3)	114 (16.0)	
Do not use	366 (15.5)	59 (16.1)	

*Facemask change*			
1–2 times	1573 (66.8)	237 (15.1)	0.920
>2 times	364 (15.4)	57 (15.7)	
Do not use	419 (17.8)	66 (5.8)	

## Data Availability

The data that support the study can be obtained from the corresponding author upon request.
